# Country profile on family medicine and primary health care in Ghana

**DOI:** 10.4102/phcfm.v8i1.1302

**Published:** 2016-11-22

**Authors:** Henry J.O. Lawson, Akye Essuman

**Affiliations:** 1 Department of Community Health, School of Public Health, University of Ghana, Ghana

## Overview of country and burden of disease

In 2010, Ghana became a lower middle-income country with a gross domestic product (GDP) of $40.12 billion ($39.20 billion in 2011) and a gross national income per capita of $1810. Despite this overall progress, there is still significant inequality in economic growth between the north and south, which translates into a gap in human development where the north lags behind. With an estimated 28.6% of the population living below the poverty line of $1.25 per day, Ghana’s total health expenditure represents 4.8% of the GDP (total expenditure on health per capita is $90 per person), and 59.5% of this is spent in the public sector.^1^ Ghana is being confronted with a double burden of disease, with non-communicable chronic diseases increasingly featuring among the top 10 diseases seen.^2^

The Ministry of Health (MoH) has the goal of providing quality, affordable and accessible health services delivered in a humane, efficient and effective manner by health professionals to improve the health status of Ghanaians.^3^

## Current primary health care system: Description, strengths and weaknesses

**FIGURE 1:**
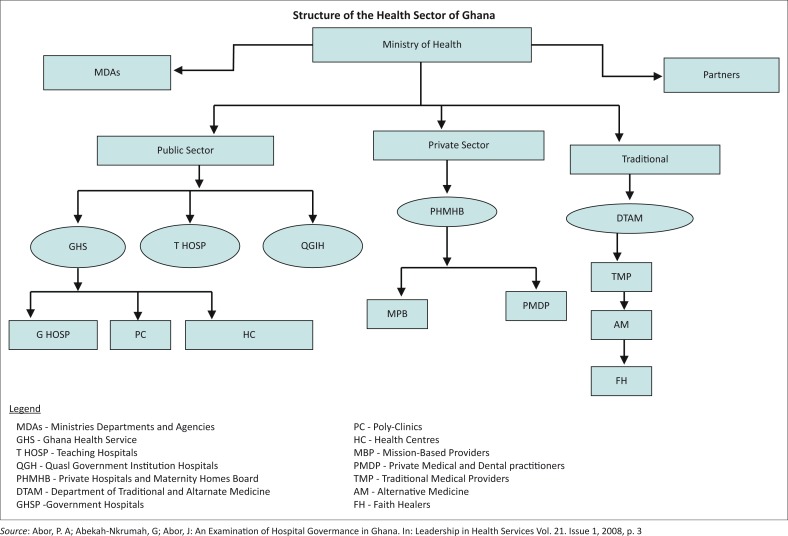
Structure of the health sector of Ghana.

With a population-to-doctor ratio of 10 032:1 and a population-to-nurse ratio of 1240:1 in 2011, Ghana’s health service delivery system is gradually shifting the focus from consultations with doctors or nurses in facilities to primary health care provision (via community health workers) at the community level in order to effectively reach remote rural populations (see Figure 1). A close-to-client service delivery plan was adopted in Ghana through the community-based health planning and services (CHPS) in 2000, where community health nurses were placed in communities to offer public health, outreach and to serve as the first point of clinical contact and referral. Each CHPS compound is supposed to serve, on average, a population of 500 and is demarcated according to the government’s 6135 electoral areas. The CHPS programme provides a wide range of essential preventive and curative services to some of Ghana’s most rural and impoverished locations. The genesis of the CHPS strategy adopted by the MoH showed that assigning nurses to community locations reduced childhood mortality rates by more than 50% in 3 years and accelerated attainment of the child survival Millennium Development Goal. Fertility was also reduced by 15%, representing a decline in birth rate. The programme cost an additional $1.92 per capita to the $6.80 per capita primary health care budget.^4^

## Strengths

Ghana has made steady progress in the health status of its population over the past few decades, with life expectancy improving from 50 years in 1960 to 62 years in 2012. Child health has improved, resulting in a decrease in child mortality rate from 115 per 1000 in 1983 to 80 in 2008, and infant mortality decreased to 50 deaths per 1000 live births during the same period. Maternal health is also improving, with a decrease in maternal deaths by 42% from 740 in 1990 to 350 deaths per 100 000 live births.^2^

Five health-related laws – *Public Health Act*, 851; *Mental Health Act*, 846; *National Health Insurance Act*, 852; *Health Institutions and Facilities (HIFA) Act*, 829; and *Specialist Health Training and Plant Medicine Research Act*, 833 – were passed by the parliament in 2011 and the *Health Professions Regulatory Bodies Act*, 857. Most of the Acts have been influenced by global or regional orientations such as World Health Assembly Resolutions.^2^

The National Health Insurance Scheme (NHIS) has a reasonably generous benefit package, covering 95% of disease conditions that afflict people in Ghana. Some of the benefits covered include out-patient services, inpatient services, oral health and maternity care. The NHIS, however, excludes the following services: appliances, prostheses, rehabilitation, dentures, organ and cosmetics surgery and assisted reproduction, HIV retroviral drugs, hormone and organ replacement therapy, heart and brain surgery other than accident, diagnosis and treatment abroad, dialysis for chronic renal failure and cancers.^5^

To encourage service in rural areas, the government has implemented special non-salary incentives to motivate nurses to stay and work at rural CHPS posts.

To fill gaps in service delivery, the government conceived and implemented another programme in 2010 to train and deploy 31 400 lower cadres of health care workers – 6400 health promotion officers (HPOs) and health promotion assistants (HPAs) and 25 000 health extension workers (HEWs) to the districts, subdistricts and communities under a public–private partnership between the MoH, Ministry of Youth and Sports and Better Ghana Management Services, a private sector company. This programme supplements and complements the efforts of the trained professionals in the CHPS zones and other health posts around the country.

## Weaknesses

Nutritional status of children however still remains a challenge, with about 28% of Ghanaian children being stunted, 9% being wasted and 14% being underweight. Financial and geographical access to health care continue to be key challenges confronting the health sector of Ghana despite the NHIS and the expansion of CHPS. Quality health service is another gap accounted for by limited skilled health workers, poor skill mix and inequity in distribution to the advantage of the south and urban areas.^2^ An important lesson learnt from the CHPS programme is that many of the trained nurses were unwilling to live and/or work in the remote areas of the country where their services are needed most. Even nurses who come from rural areas became reluctant to leave urban living situations and refused to return to the villages.

## Current place of family medicine in the health system

Family physicians in Ghana are trained under two independent colleges: the West African College of Physicians (WACP) and Ghana College of Physicians and Surgeons (GCPS). There are two levels of training after passing the entry examination and interview: membership and fellowship. A member requires a minimum of 3 years training and an exit examination, making a total of approximately 5 years post-MBChB qualification. Fellowship training requires an additional 2–3 years. Members are integrated into the health care system as specialist family physicians. They have been used variously as clinical heads and medical superintendents in district hospitals. This position is equivalent in remuneration and recognition to internists, laboratory physicians, public health physicians, paediatricians and psychiatrists. A fellow is integrated into the public health care systems as a senior specialist similar to the specialties listed above. Currently, 6 senior specialists and 35 specialists are working in various positions across the country. About 15% are in the private, military and quasi-government health facilities. In a bid to accelerate the rate of training of family physicians, a new modular family medicine training programme has been inaugurated by the Faculty of Family Medicine of the GCPS. The Ghana Health Service and the GCPS have signed a memorandum of understanding to work together in a bid to decentralise specialist training programmes in the districts. Under this initiative, doctors no longer have to leave their positions in the district, especially in rural communities, to pursue postgraduate training. Family medicine has been selected to pilot this programme in the country.

## Current state of family medicine in the educational system

Family medicine in its current form was introduced in Ghana through the efforts of some general practitioners who were members of the Society of Private Medical and Dental Practitioners of Ghana. In the early 1990s, these experienced practitioners were formally elected as foundation fellows of the Faculty of General Medical Practice (GMP) of the WACP, Ghana chapter, and charged with the responsibility of initiating postgraduate training in general practice in Ghana. The WACP residency programme officially began in April 1999 with three candidates. In 2005, the GCPS began a national residency programme in family medicine with two candidates in the two main teaching hospitals in Accra and Kumasi. Doctors who apply for this training should have completed their basic medical degree (MBChB or its equivalent) and completed 2 years of housemanship. In addition, they need to have worked in a district for at least 1 year. The training is in three parts for both colleges. There is an entry examination with/without an interview, a membership level examination after 3 years of training and a fellowship level examination after a further minimum of 2 years of training. At the time of writing, 36 family physicians have been trained and 29 junior residents were in training.

## Undergraduate family medicine in Ghana

Various unsuccessful attempts were made since 1989 to run an undergraduate programme through the establishment of an academic department of family medicine. In early 2007, two fellows of the specialty were appointed as lecturers in the University of Ghana Medical School to develop an appropriate curriculum for an undergraduate programme and promotion of the specialty through research. The specialty currently functions as a unit in the Department of Community Health. In July 2008, a survey was conducted among the very first batch of students to be taught the principles of family medicine to determine their perceptions about family medicine with regard to knowledge and relevance as well as specialty preferences. The level of awareness of the specialty was high among the students (88.0% [95% confidence interval (CI) 80.2–93.6]). Majority of the students perceived family physicians as being capable of providing total health care for 85–95% of their clients (54.4%, CI 44.1–64.5) and also reducing overall cost of health care (79.8%, CI 70.5–87.2). However, only 2.4% (CI 0.4–7.6) were considering postgraduate training in family medicine, and the main reason for not choosing family medicine was inadequate understanding of the specialty (79.3%, CI 69.5–87.0). The study therefore concluded, among others, that it was expedient to offer the opportunity for early exposure of medical students to the principles of family medicine.^6^
